# Renin-Angiotensin-Aldosterone System Blockade (RAASB) After Acute Kidney Injury: The Controversy Thickens on If and When to Discontinue RAASB

**DOI:** 10.7759/cureus.49571

**Published:** 2023-11-28

**Authors:** Macaulay A Onuigbo

**Affiliations:** 1 Medicine, The Robert Larner, M.D. College of Medicine, University of Vermont, Burlington, USA

**Keywords:** late onset renal failure from angiotensin blockade (lorffab), angiotensin receptor blocker (arb), ace inhibitor (acei), chronic kidney disease (ckd), acute kidney injury (aki), renin-angiotensin-aldosterone system blockade (raasb)

## Abstract

Unquestionably, there is a common consensus regarding cardiorenal protection with renin-angiotensin-aldosterone system blockade (RAASB) in both diabetic and nondiabetic chronic kidney disease (CKD). Nevertheless, there remain conflicting retrospective reports regarding renal and cardiovascular mortality outcomes following discontinuation of RAASB in advanced CKD. We present an editorial on a recent article discussing renal and mortality outcomes among hospitalized veterans who were started back on RAASB versus those who were not started back on RAASB. The controversy surrounding this topic thickens as the analysis unfolds.

## Editorial

We read with exaggerated anticipatory excitement the just-published article by Murphy et al. on renin-angiotensin-aldosterone system blockade (RAASB) use after acute kidney injury (AKI) in 54,735 hospitalized veterans [1]. In this retrospective analysis of a very large veterans population, the authors demonstrated lower mortality associated with the use of angiotensin-converting enzyme inhibitors/angiotensin receptor blockers (ACEIs/ARB) < three months after hospitalization in veterans with diabetes, proteinuria, and AKI, regardless of renal recovery status [1]. AKI in this study was rigorously defined as an increase in serum creatinine of ≥50% above baseline [1]. Significantly, 95% of the participants had a baseline estimated glomerular filtration rate (eGFR) of ≥30 ml/min/1.73 m^2^, and 65% of the participants had a baseline eGFR of ≥60 ml/min/1.73 m^2 ^[1].

Unquestionably, there is a common consensus regarding cardiorenal protection with RAASB in both diabetic and nondiabetic CKD. Nevertheless, there remain conflicting retrospective reports regarding renal and cardiovascular mortality outcomes following discontinuation of RAASB in advanced CKD [2,3]. In a large Swedish Registry observational study of people with advanced CKD, stopping RAASB was associated with higher absolute risks of mortality and major adverse cardiovascular events, but with a lower risk of kidney replacement therapy [2]. Most pertinently, over 15 years ago, we had first described the syndrome of late-onset renal failure from angiotensin blockade (LORFFAB), and we recently replicated findings consistent with LORFFAB in an article published in the *Cureus *journal [4,5]. In the 2022 *Cureus* journal report, 71 patients who presented with otherwise inexplicable new-onset and progressive AKI while on RAASB, in general, had demonstrated sustained renal recovery, and this was without increased cardiovascular mortality, following the preemptive discontinuation of RAASB [5]. Death in 11 of 12 (91%) of our patients was from non-renal causes, and most deaths occurred despite improved kidney function [5].

While we strongly associate with the findings of Murphy et al., we also agree with the authors that “finally, residual confounding in our study is possible despite multiple adjustors” [1]. We posit that it is indeed possible that the undocumented reasons for the non-continuation of RAASB in the veterans population may have reflected patients with more severe AKI during hospitalization. Furthermore, in the study by Murphy et al., 95% of the participants had baseline eGFR ≥ 30 ml/min/1.73 m^2^, and 65% had eGFR ≥ 60 ml/min/1.73 m^2 ^[1]. It is, therefore, possible that in much older patients with much lower baseline eGFR, and with more severe AKI at presentation, discontinuation and subsequent non-continuation of RAASB for these unmeasured reasons represented the unmeasured confounding variables in the veterans study. Similarly, Fu et al. acknowledged that the precise reasons for stopping RAASB in their study remained unknown and may have impacted study outcomes as an unknown confounding factor [2]. To further support our deposition that in patients on RAASB who developed new-onset, progressive, and otherwise inexplicable declining kidney function, the withdrawal of RAASB should be the standard next step. Seeman and Dušek, in a recent study, demonstrated that the withdrawal of ACEIs in seven children with CKD stage 4-5 and rapidly declining kidney function led to an increase in eGFR after a median follow-up of 2.7 (range: 0.5-5.0) years [6]. Similarly, Ahmed et al. had previously demonstrated increased eGFR in 60% of adults with advanced CKD and had confirmed undoubtedly delayed onset of kidney replacement therapy in the majority of patients after stopping inhibitors of the renin-angiotensin-aldosterone system [7].

In conclusion, we have long posited that in patients presenting with new-onset and progressive acutely worsening AKI, in the absence of any other plausible explanation for the AKI, a trial withdrawal of RAASB remains an electable, defensible, and reasonable therapeutic option. A well-developed randomized controlled trial (RCT) study of this hypothesis has so far not been completed. The completion of such a study is long overdue.
